# Neuroanatomy of the Vestimentiferan Tubeworm *Lamellibrachia satsuma* Provides Insights into the Evolution of the Polychaete Nervous System

**DOI:** 10.1371/journal.pone.0055151

**Published:** 2013-01-23

**Authors:** Norio Miyamoto, Ayuta Shinozaki, Yoshihiro Fujiwara

**Affiliations:** 1 Institute of Biogeosciences, Japan Agency for Marine−Earth Science and Technology, Yokosuka, Japan; 2 Graduate School of Biosphere Science, Hiroshima University, Higashi-Hiroshima, Japan; Sars International Centre for Marine Molecular Biology, Norway

## Abstract

Vestimentiferan tubeworms are marine invertebrates that inhabit chemosynthetic environments, and although recent molecular phylogenetic analyses have suggested that vestimentiferan tubeworms are derived from polychaete annelids, they show some morphological features that are different from other polychaetes. For example, vestimentiferans lack a digestive tract and have less body segments and comparative neuroanatomy can provide essential insight into the vestimentiferan body plan and its evolution. In the present study, we investigated the adult nervous system in the vestimentiferan *Lamellibrachia satsuma* using antibodies against synapsin, serotonin, FMRMamide and acetylated α-tubulin. We also examined the expressions of neural marker genes, *elav* and *synaptotagmin* to reveal the distribution of neuronal cell bodies. Brain anatomy shows simple organization in *Lamellibrachia* compared to other polychaetes. This simplification is probably due to the loss of the digestive tract, passing through the body between the brain and the subesophageal ganglion. In contrast, the ventral nerve cord shows a repeated organizational structure as in the other polychaetes, despite the absence of the multiple segmentation of the trunk. These results suggest that the brain anatomy is variable depending on the function and the condition of surrounding tissues, and that the formation of the rope ladder-like nervous system of the ventral nerve cord is independent from segmentation in polychaetes.

## Introduction

Vestimentiferan tubeworms are marine invertebrates that live in chemosynthetic environments such as hydrothermal vents and hydrocarbon seeps. They have a unique body plan as they lack a digestive system, including a mouth and anus. Instead of the typical digestive system, they harbor symbiotic chemoautotrophic bacteria in an internal organ, the trophosome, and derive their metabolic needs from these bacteria [1], [2] (and reviewed in [3]). The body of vestimentiferan tubeworms consists of four regions: the tentacular region, the vestimental region, the trunk, and the opisthosome. The opisthosome is the only multi-segmented region. Due to the unique morphological features of vestimentiferans, they were previously classified into the independent phylum Vestimentifera [4]. Although the phylogenetic position of vestimentiferans has been controversial for a long time [5], [6], recent morphological and molecular phylogenies have strongly supported that they are modified polychaetes and they have subsequently been assigned to the family Siboglinidae together with franulates, moniliferans and *Osedax* [7]−[15]. Their unique morphological features have fascinated scientists: however, our knowledge of the body plan of vestimentiferans and its origin is limited, largely because of poor accessibility to specimens for examination.

**Figure 1:**
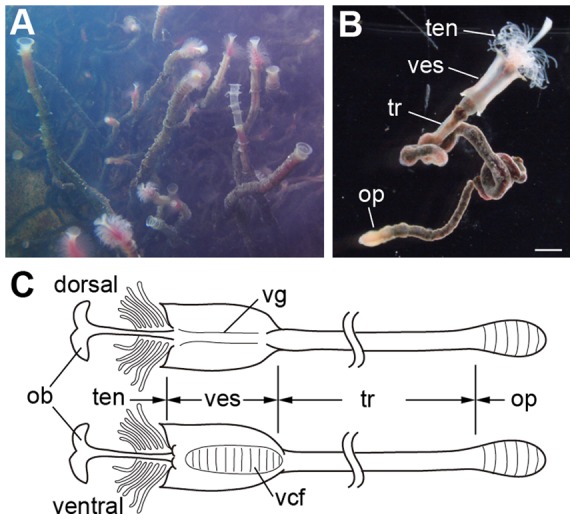
Anatomical overview of *L. satsuma*. **A:** Colony of *L. satsuma* attached on a whale bone in an aquarium. **B:** A worm removed from a tube shows four body regions. **C:** Dorsal (upper) and ventral (lower) views. Scale bar: **B:** 1 mm. ob, obturaculum; op, opisthosome; ten, tentacular region; tr, trunk; vcf, ventral ciliated field; ves, vestimental region; vg, vestimental groove.

Study of the neuroanatomy provides a better understanding of the evolution of the animal body plan (e.g., [16]). The nervous system of polychaetes has been well studied [17]−[20] and consists of the brain, the ventral nerve cord, and the peripheral nervous system. The brain and the ventral nerve cord are connected through the circumesophageal connectives, and the ventral nerve cord shows a rope ladder-like organization that reflects the segmented body plan. This plan is highly conserved among polychaetes, although minor modifications occur in some taxa. Compared to the other polychaetes, observations of the vestimentiferan nervous system have been limited to histological and ultrathin sectioning [21]−[24]. In the present study, we examined the neuroanatomy of *Lamellibrachia satsuma* Miura, Tsukahara and Hashimoto, 1997 (Figure 1) by immunohistochemistry and *in situ* hybridization of nervous system markers to elucidate how the loss of the digestive system and body segments affects the organization of the nervous system. We found that its brain anatomy is distinct from other polychaetes, whereas the ventral nerve cord consists of a rope ladder-like organization with repeated ganglia. This result suggests that the rope ladder-like ventral nerve cord is independent from the segmentation.

## Results

### Brain

**Figure 2:**
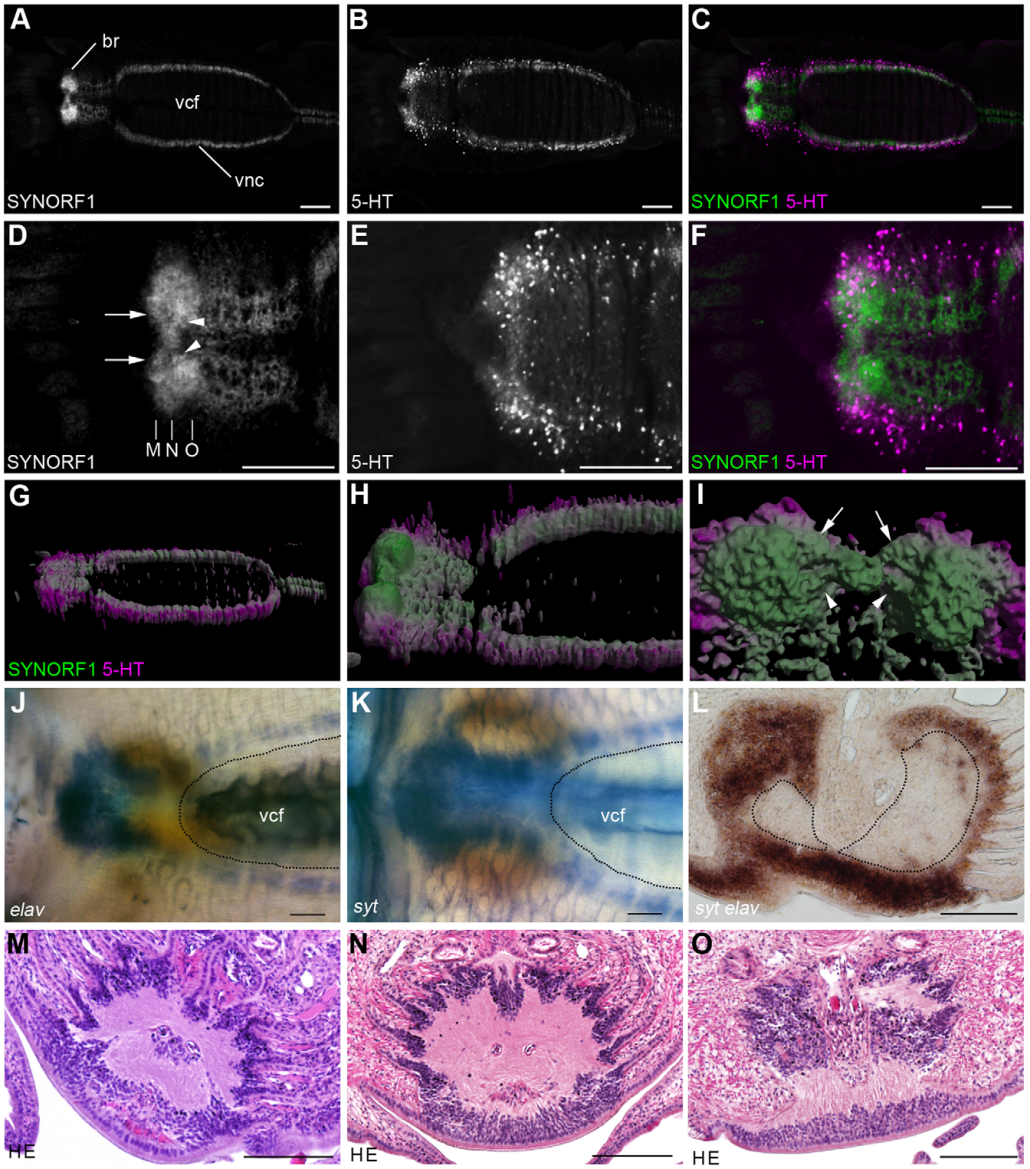
Brain and ventral nerve cord of the tentacular and vestimental regions in *L. satsuma*. **A–C:** Z-projection images of anti-SYNORF1 and anti-serotonin immunoreactivities showing Anti-SYNORF1 positive neuropils and serotonergic neurons of the brain and the ventral nerve cord. **D–F:** Z-projection images of anti-SYNORF1 and anti-serotonin immunoreactivities at the anterior end of the central nervous system. A pair of prominent neuropils is connected through the anterior (arrows) and posterior (arrowheads) connectives. **G–I:** Three-dimensional reconstructions of the central nervous system. Ventrolateral view (G) and dorsolateral view (H) views show that anti-SYNORF1 positive neuropils in the brain are thickened dorsally. Serotonergic neurons are located on the ventral and lateral sides of the anti-SYNORF1 positive neuropils. **I:** Dorsal view of the brain: anterior is up. The anterior (arrows) and posterior (arrowheads) anti-SYNORF1 positive signals connect a pair of neuropils. **J, K:** Expression patterns of *elav* (J) and *syt* (K) showing the distribution of neural cell bodies. **L:** Section through the posterior end of the brain neuropils. Dorsal is up. Double stain of *syt* (brown) and *elav* (yellow) shows the overlapped expression patterns of these neuron marker genes. The neuropils are encapsulated by neurons. **M−O:** Cross sections of brain. Positions are shown in **D**. The cell bodies cover the neuropils. Scale bars: 100 µm. br, brain; vcf, ventral ciliated field.

The brain of *L. satsuma* is situated at the ventroanterior position in the vestimentum where the diverged ventral nerve cord meets at the ventral midline (Figure 2A–C). Anti-SYNORF1 immunoreactivity showed that neuropils are present bilaterally in the brain (Figure 2D). The two SYNORF1 positive signals, which are probably the commissures, connect the neuropils at the midline of the brain (Figure 2D, I). Serotonergic neurons are located on the ventrolateral side of the brain (Figure 2E, F). The three-dimensional reconstructions of the brain showed that the neuropils of the brain are thickened dorsally, whereas the ventral nerve cord runs just under the ventral epidermis (Figure 2G–I). To reveal the distribution of the cell bodies of the neurons, we examined the expression patterns of two neural marker genes, *syt* and *elav*. The expressions of these marker genes were detected in the brain and in the ventral nerve cord (Figure 2J, K). The cross section of the brain showed that neurons are present just under the thickened epidermal cells (Figure 2L). The expressions of *syt* and *elav* were also detected in the laterally and dorsally encapsulated neuropils of the brain (Figure 2L). The expressions of neuron marker genes evenly covered the neuropil and no distinct cluster of neurons, such as a mushroom body, was found in the brain. The cell bodies and neuropils of the brain were observed by hematoxylin and eosin (HE) staining (Figure 2M−O). Eosin stained neuropils are covered by hematoxylin-stained cell bodies.

### Ventral nerve cord

**Figure 3:**
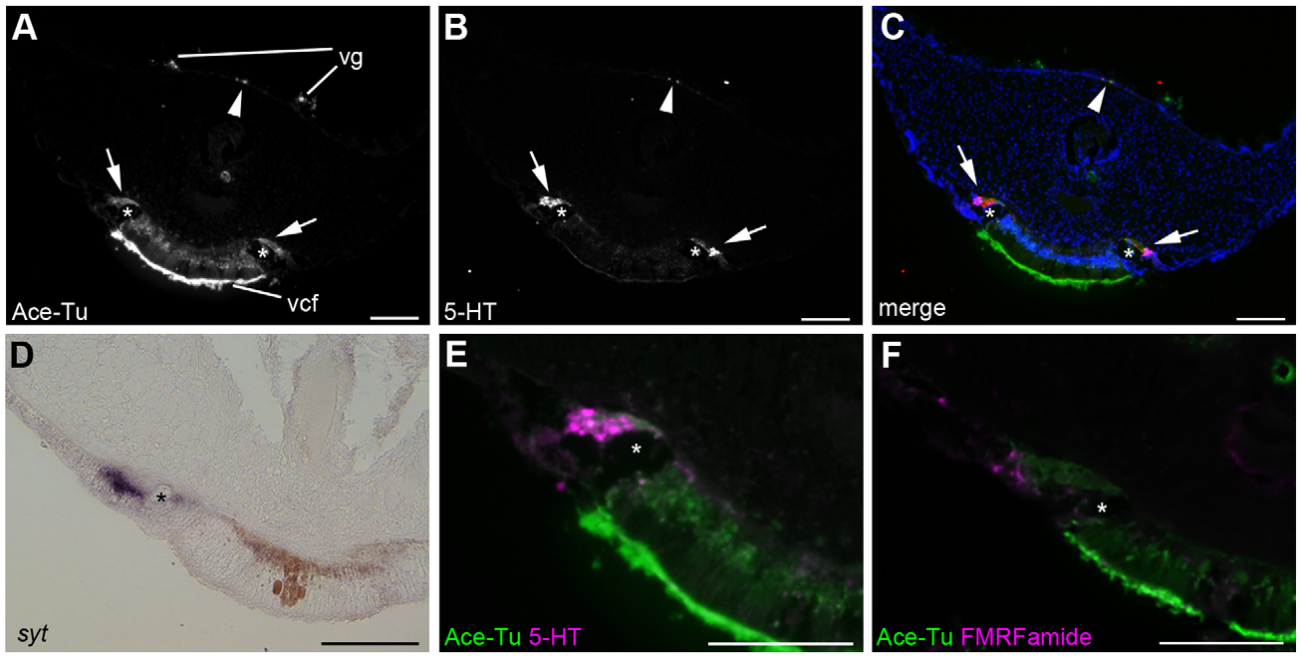
Ventral and dorsal nerve cords in the vestimental region. **A–C, E:** Anti-acetylated tubulin and anti-serotonin immunoreactivities. Serotonergic neural cell bodies are located on the lateral side of the giant axon (asterisks), and they project their axons medially (arrows). Anti-serotonin immunoreactivity was found in the dorsal nerve cords (arrowheads). **D:** Expression of *syt* was detected in the ventral nerve cord. **F:** Double-stain against anti-acetylated tubulin (green) and anti-FMRFamide (magenta) antibodies shows the presence of FMRFamidergic neurons in the ventral nerve cord. Scale bars  = 100 µm. vcf, ventral ciliated field; vg, vestimental groove.

Anti-acetylated tubulin and anti-serotonin immunoreactivities showed that the ventral nerve cord, which runs through the ventral midline of the trunk and the opisthosome, is continuous from the brain (Figure 2A–C). In the vestimental region, the ventral nerve cord is separated into two rows and each row runs along the lateral boundary of the ventral ciliated field (Figure 3A–C). The expression of *syt* indicated neural cell bodies located on the lateral side of the giant axon, a distinct tubular structure in the vestimentiferan nervous system (Figure 3D). In addition to *syt*, *elav* was also expressed on the lateral side of the giant axon (data not shown). Neurons project axons to the dorsal side of the giant axon (Figure 3A–C, asterisks). Serotonergic and FMRFamidergic neurons are present in the ventral nerve cord of the vestimental region (Figure 3E, F).

**Figure 4:**
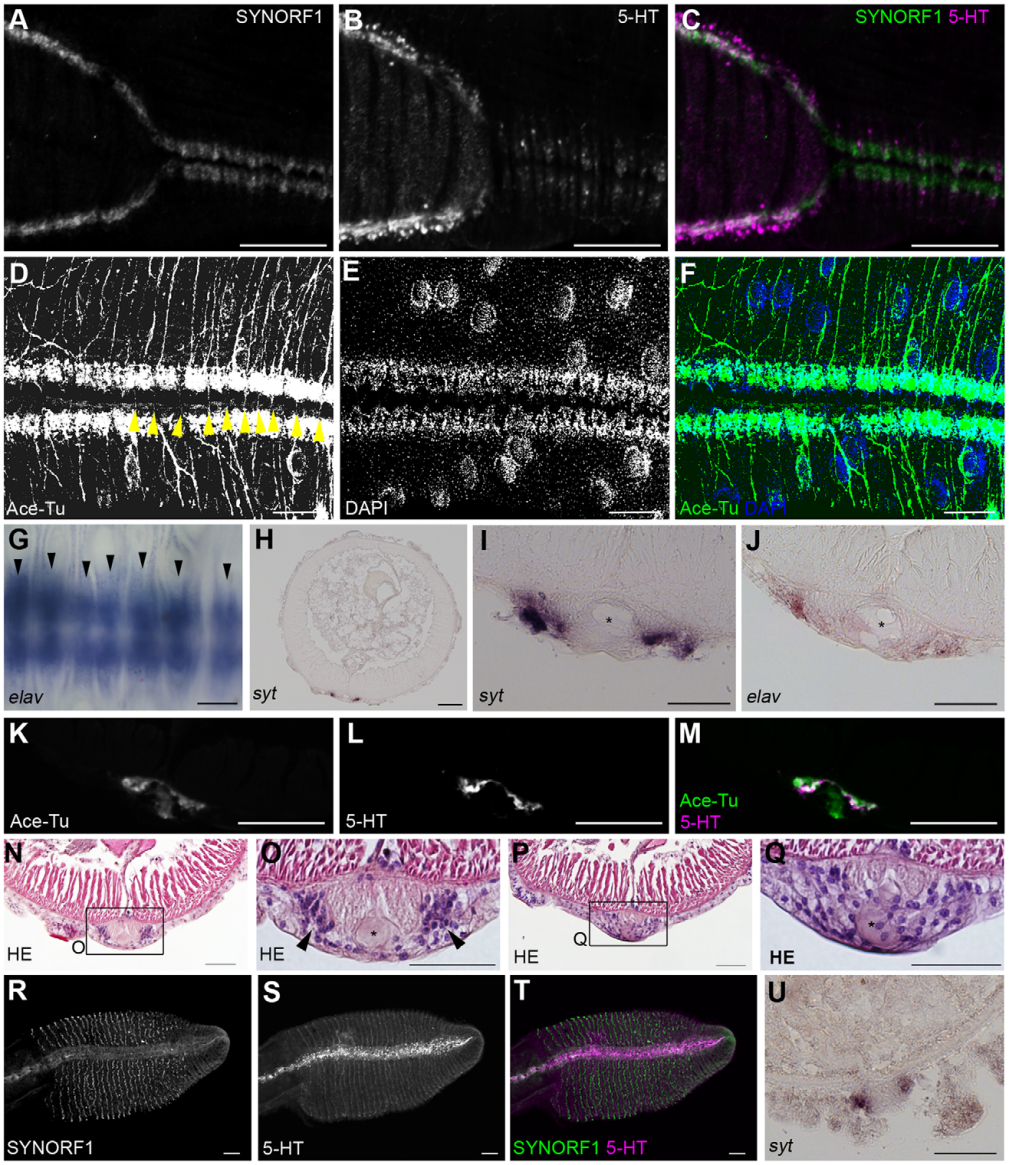
Ventral nerve cord in *L. satsuma*. **A–C:** Double stain against SYNORF1 (green) and anti-serotonin (magenta) immunoreactivities from the posterior end of the vestimental region to the anterior end of the trunk shows localizations of neuropil and serotonergic neurons of the ventral nerve cord. **D–F:** Double stain with anti-acetylated tubulin (green) and DAPI (blue) of the anterior part of the ventral nerve cord in the trunk shows the metameric organization of the nerve cord. The anterior is shown on the left. Yellow arrowheads indicate commissures. **G:** Ventral view of whole-mount image of *elav* expression showing the repeated pattern of neurons (arrowheads). The anterior is on the left. **H–J:** Sectioned images of *syt* (H, I) and *elav* (J) expressions. Neurons are located on the lateral side of the ventral nerve cord. Asterisks indicate the position of giant axon. **K−N:** Sections of the ventral nerve cord at positions of ganglia (K, L) and of inter ganglia (M, N) **O–Q:** Anti-acetylated tubulin (green) and anti-serotonin (magenta) immunoreactivities in a cross section of the ventral nerve cord show ventrally located serotonergic neurons and dorsally located axons. **R–T:** Double stain against SYNORF1 (green) and anti-serotonin (magenta) immunoreactivities in the opisthosome shows that the localization of serotonergic neurons in the ventral nervous system of the opisthosome. **U:** Cross section of the opisthosome shows *syt* expression in the ventral nerve cord. Scale bars: A–H, O–T  = 100 µm; I−N, Q = 50 µm.

In the posterior end of the vestimental region, two nerve cords meet at the ventral midline and merge to form a single nerve cord (Figure 4A–C), as previously observed [17]. In the anterior part of the trunk, the ventral nerve cord shows a repeated organizational structure, whereas no segmentation occurs in the trunk (Figure 4D–F). Ganglion-like cell masses and anti-acetylated tubulin immunoreactivity were repeatedly found, and axons projected laterally from each cell mass. Commissure neurons connecting each side of the nerve cord were also found (Figure 4D, arrowheads). The expression pattern of *elav* was metameric (Figure 4G), as was the *syt* expression (data not shown). These findings show that the ventral nerve cord of *L satsuma* is essentially a rope ladder-like nervous system, as found in other annelids. The expressions of neuron marker genes and cross sections of anti-acetylated tubulin and serotonin immunoreactivities showed that cell bodies of neurons are located on the ventrolateral sides of the ventral nerve cord, and axons run along the dorsomedial parts of the ventral nerve cord (Figure 4I–M). The is the single giant axon occurs in the middle of the nerve cord (Figure 4I, J, asterisks). The ganglionic and interganglionic regions were detected by histological staining. Sections at the level of the ganglion showed a pair of cell masses (Figure 4N, O). In contrast, no such cell masses were observed at the level of the interganglionic regions, and cells showed a scattered distribution (Figure 4P, Q).

The ventral nerve cord extends to the posterior end of the opisthosome (Figure 4R–U). In the opisthosome, serotonergic neurons project their axons laterally along the segments (Figure 4R, T). The expression of *syt* showed that cell bodies of neurons located on the lateral sides of the cord and the giant axon are not present in this region (Figure 4U). As previously described in *Lamellibrachia barhami* [25], the ventral nerve cord in *L. satsuma* is an intraepidermal nerve extending from the vestimental region to the opisthosome (Figures 3, 4).

### Dorsal nerve cord

**Figure 5:**
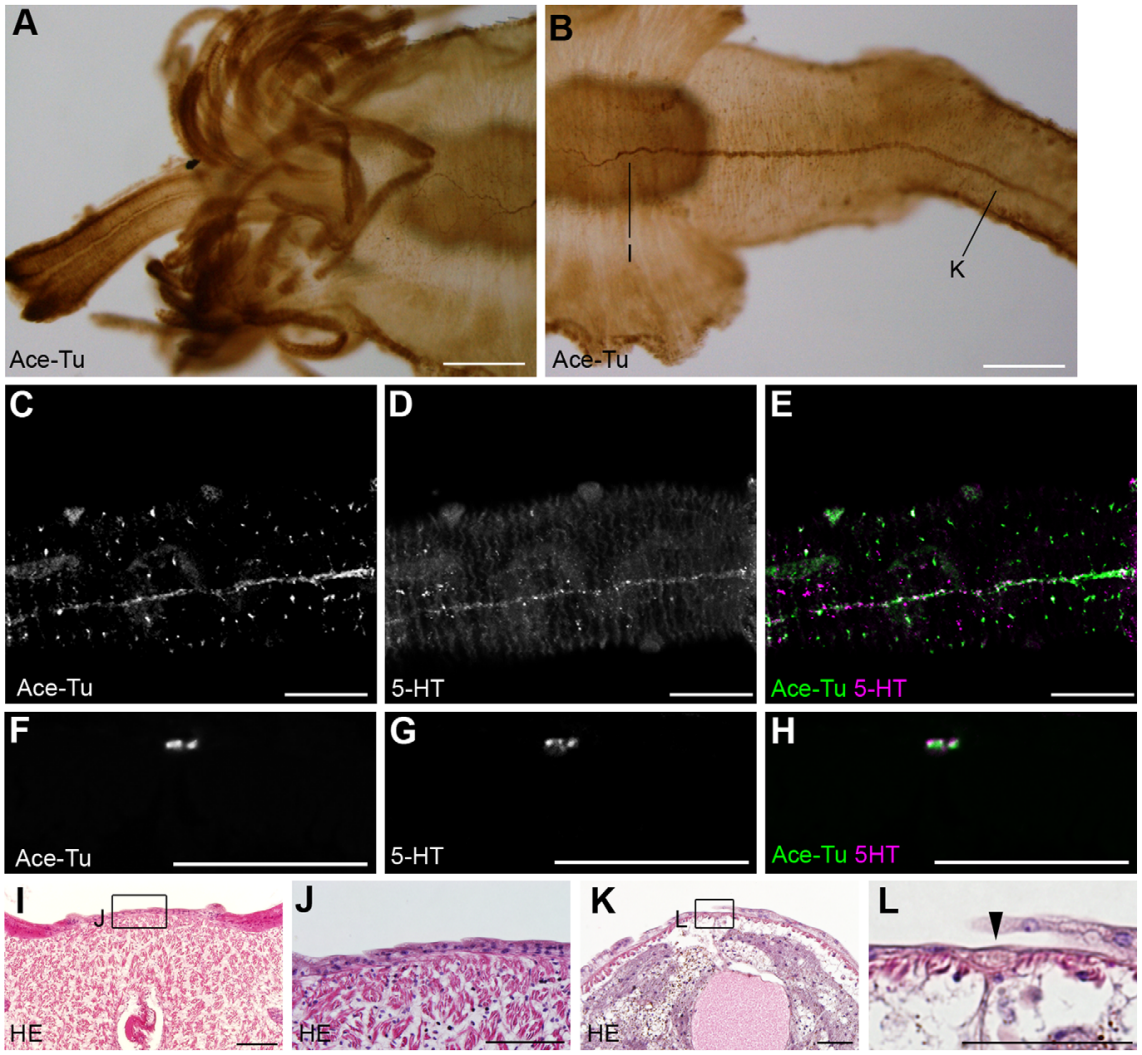
Dorsal nerve cord in *L. satsuma*. **A, B:** Immunoreactivities against anti-acetylated tubulin antibody show that the dorsal nerve cord is continuous from the vestimental region to the trunk. The cord branches in the anterior half of the vestimental region. **C–H:** Double stain against anti-acetylated tubulin (green) and anti-serotonin (magenta) antibodies shows that the dorsal nerve cord of the trunk is a serotonergic and non-metameric cord. **C–E:** Confocal laser scanning microscopy of the whole animal. **F–H:** Cross section of the trunk. **I, J:** Cross section of the vestimentum. No axon of the dorsal nerve cord was detected by HE staining. **K, L:** Cross section of the trunk showing the thin fiber of the dorsal nerve cord. Scale bars: A, B  = 200 µm; C–H  = 100 µm; I–L  = 50 µm.

We found anti-acetylated tubulin and anti-serotonin immunoreactivities in the dorsal midline and consider it a dorsal nerve cord. The dorsal nerve cord starts in the anterior one-third of the vestimentum and extends to the end of the opisthosome at the dorsal midline (Figure 5). At the anterior end, the dorsal nerve cord divides into several branches (Figure 5A, B). We could not observe any connection between the dorsal nerve cord and the brain. Immunoreactivity of the anti-serotonin antibody showed that the dorsal nerve cord consists of serotonergic neurons (Figure 5C–H). We could not detect any repeated structure in the dorsal nerve cord. No expression of the neuron marker genes was detected in the dorsal nerve cord (data not shown). In the vestimentum, the dorsal nerve cord was not detected by histological staining (Figure 5I, J) due to the very thin fibrous nature of the cord. In the trunk, the dorsal nerve cord was detected as a thin intraepidermal nerve (Figure 5K, L).

### Sensory structure around the pyriform gland

**Figure 6:**
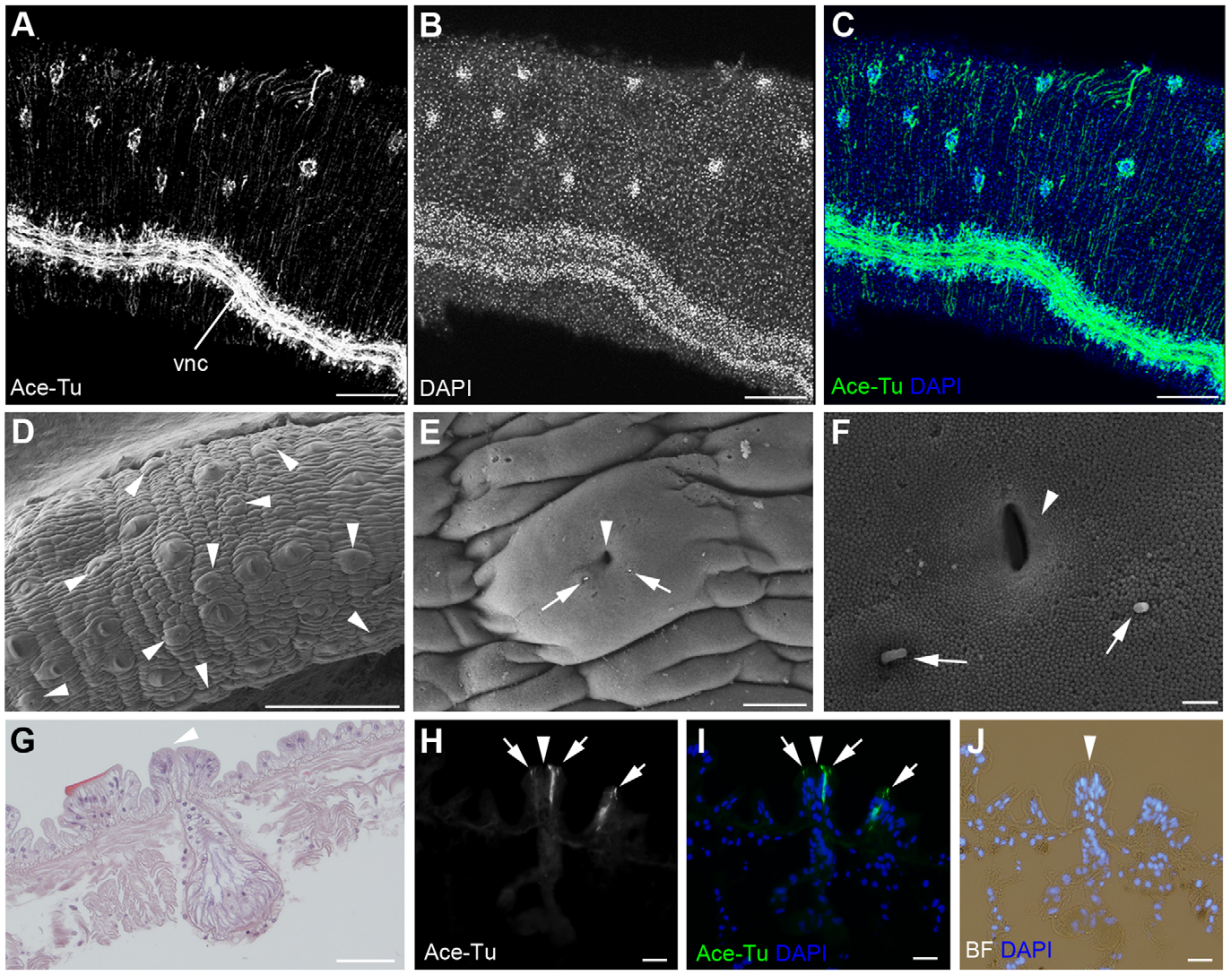
Peripheral sensory neurons in the trunk. **A–C:** Immunoreactivity against anti-acetylated tubulin antibody (green) and DAPI staining (blue) of the posterior part of the trunk shows the immunoreactive cell masses on the lateral side of the trunk. **D:** SEM images of the lateral side of the trunk showing the number of pyriform glands in the trunk (arrowheads). **E, F:** High-magnification images of a pyriform gland show a pore of the gland (arrowhead) and two cilia near the pore (arrows). **G:** A HE-stained section of a pyriform gland. Arrowhead indicates a pore of the gland. **H–J:** Anti-acetylated tubulin immunoreactivity for a cross-section of the pyriform gland showing two immunoreactive cells (arrows) near the pore of the gland (arrowhead). Scale bars: A–D  = 200 µm; E = 10 µm; F = 1 µm; G = 50 µm; H–J  = 20 µm. vnc, ventral nerve cord.

We found sensory structures in the trunk region. Anti-acetylated tubulin immunoreactivities were observed on the lateral surface of the trunk (Figure 6A). A double stain with DAPI showed that cell masses are present within the anti-acetylated tubulin immunoreactive cells (Figure 6B, C). SEM observation indicated that the pyriform glands are distributed in the same manner (Figure 6D, arrowheads) (i.e., the anti-acetylated tubulin immunoreactive cell mass is the pyriform gland). The gland has a pore at the center with two cilia beside the pore (Figure 6E, F, arrowheads and arrows). The pyriform gland is a bottle-shaped and secretion cells with prominent vacuoles are located at the bottom of the hole (Figure 6G). Two anti-acetylated tubulin immunoreactive cells are found in the each gland (Figure 6H–J). These observations demonstrated that the pyriform gland has sensory cilia, which may be related to the secretory function of the gland.

## Discussion

The neuroanatomy of *L. satsuma* was examined in detail by immunohistochemistry and *in situ* hybridization. We found that the brain anatomy of *L. satsuma* is dramatically different from that of other polychaetes. The modification reflects the evolutionary changes in the head morphology of *L. satsuma*. In polychaetes with a distinct digestive tract, the anterior part of the central nervous system consists of the brain, or supraesophageal ganglion, and the subesophageal ganglion [17], [18]. These two prominent ganglia are connected by circumesophageal connectives, which encircle the esophagus. In contrast, vestimentiferan tubeworms have lost their digestive tract. Previous studies have shown that vestimentiferans have a functional digestive tract in their early developmental stage [26]. The digestive tract degenerates during metamorphosis, and a remnant passes through the brain [26]. This extensive modification of the head structure may affect the anatomy of the brain of vestimentiferans. The brain of *L. satsuma* is located on the ventral side, whereas those of the other polychaetes are on the dorsal side. We could not find distinct structures of the brain, subesophageal ganglion and circumesophageal connectives, which seem to fuse into one large ganglion called the brain in this paper. To understand how the head structures evolved, we must compare the development and expression patterns of marker genes for each tissue and the brain domains in vestimentiferans with those of the other polychaetes. The typical polychaete brain consists of several ganglia [17], the most prominent one being the central ganglion located along the anterior midline of the brain. The dorsal and commissural ganglia are found in most of the taxa [17]. Antibody staining and expression patterns of neuron marker genes showed that neurons cover the neuropils equally rather than forming clusters in *L. satsuma*. Only small masses of neurons at the basal parts of the tentacles are found in the *L. satsuma* brain. We did not observe a mushroom body in the brain, which is a notable structure in vagile polychaetes [27]−[30]. The simple structure of the *L. satsuma* brain is probably due to the sessile lifestyle of this worm. These types of reduction in neuroanatomical complexity have also been observed in sedentary polychaetes [27]. Similar regressive evolution of the nervous system has been observed in sessile, semi-sessile, and parasitic animals whose life environment and mobility are simplified [31], [32].

The ventral nerve cord of *L. satsuma* shows repeated organization as in other polychaetes, despite the absence of the repeated segments in the trunk region. The presence of a rope ladder-like ventral nerve cord has been regarded as the plesiomorphic condition for annelids [18]. Although the basic organization of the rope ladder-like system is conserved, recent reports have demonstrated a great diversity in the neuroanatomy of the ventral nerve cord [17], [18]. Some taxa have numerous commissures per segment, with some having up to five longitudinal nerve cords. Despite the diversity of the ventral nerve cords, the correlation between the repeated organization of the ventral nerve cord and the segmented body is conserved. The repeating pattern in the ventral nerve cord of *L. satsuma* suggests that the presence of independent mechanisms for the formation of the patterned nervous system and that of the body segments. This hypothesis is also supported by the presence of the repeated organization of the ventral nerve cord in annelids without distinct body segments, such as echiurans and myzostomid polychaetes [33], [34]. Repeated patterns of the nervous system are also observed in the neurogenesis of sipunculus [35]. These findings indicate that the repeated organization is a primitive state and a robust system that is retained even with the loss of segmentation.

In addition to the brain and the ventral nerve cord, we found the longitudinal nerve cord at the dorsal midline, i.e., the dorsal nerve cord. The presence of the peripheral longitudinal nerves is common in polychaetes (reviewed in [17]). The position and number of the peripheral longitudinal nerves vary among species. The longitudinal nerves were found ventrolaterally, laterally, and dorsally in larvae as well as in adults [17]. In *L. satsuma*, we found a single cord along the dorsal midline. Unlike the ventral nerve cord, we could not find a perikaryon in the dorsal nerve cord. In addition the present method detected no connection between the dorsal nerve cord and the brain. To understand the function of the dorsal nerve cord, further studies on the position and distribution of cell bodies, and the identification of neural cell types and innervation patterns is necessary. In addition, to elucidate the evolution of the dorsal nerve cord, detailed comparisons of the development and function of peripheral longitudinal nerves in annelids are desirable.

Müller hypothesized that the ground pattern of the annelid nervous system as follows [18]: “(1) primarily paired circumesophageal connectives with similar dorsal and ventral roots with corresponding roots interconnected via 1 dorsal and 1 ventral commissure (4 cerebral commissures); (2) a ventral nerve cord with primarily 5 connectives: 1 unpaired medial and paired paramedian and paired main connectives; (3) numerous commissures per segment; (4) numerous segmental nerves per segment; (5) a peripheral nervous system with several nerves that, apart form the dorsomedian one, occur in pairs”. Based on the hypothesis, vestimentiferans have many evolutionary modifications. The anatomy of the anterior central nervous system is dramatically modified as discussed above. In addition, *L. satsuma* has two main connectives for the ventral nerve cord and the single dorsal nerve cord. They have a single commissure per ganglion. However, it is difficult to evaluate the number of commissures because the trunk of vestimentiferans is not multi-segmented. To elucidate the evolutionary origin of the nervous system and the body plan of vestimentiferans, comparative morphological and developmental biological approaches based on dependable phylogenetic relationships are necessary. A phylogenomic analysis showed that siboglinid polychaetes are derived from sedentarian polychaetes [36]. Among sedentarians, even though the placement of siboglinids is still debated, some reports suggest that siboglinids are likely affiliated with the oweniid polychaetes [37], [38]. Siboglinids and oweniids share a neuroanatomical feature wherein the ventral nerve cords of both taxa are an intraepidermal nerve cord [39]. Another report favored Sabellida as their sister taxon [40]. Based on the morphological and molecular data, frenulates are a basal group among siboglinids, and vestimentiferans and moniliferans form the crown group as sister taxa [14], . Molecular data suggest that *Osedax* falls between frenulates and the vestimentiferan/moniliferan clade [8]. Comparative approaches between the present data and future studies of the neuroanatomy and neurogenesis of siboglinids and sabellimorphs should provide essential information on the evolution of this group. In addition, the present study on the neuroanatomy of adult vestimentiferans provides fundamental information for further studies on the function and physiology of the vestimentiferan nervous system. These studies should elucidate how these animals have adapted to living in extreme environments such as hydrothermal vents and seeps.

## Materials and Methods

### Animals

*Lamellibrachia satsuma* specimens attached to a whale vertebra were collected from a depth of 101 m in Kagoshima Bay and maintained in a laboratory as previously described [44]. For the following experiments, we used small individuals woth a vestimentum less than 1 cm in length. For each experiment, we observed at least three individuals.

### Immunohistochemistry

For immunohistochemistry, the animals were fixed with 4% paraformaldehyde (PFA) in MOPS buffer (0.1 M 3-(N-morpholino) propanesulfonic acid, 0.5 M NaCl) at room temperature (RT) for 30 min, then rinsed twice in phosphate-buffered saline (PBS; 0.1 M, pH 7.4). Animals were stored in PBS containing 0.1% NaN_3_ in a refrigerator. Both whole body animals and frozen sectioned specimens (10 µm in thickness) were stained as follows. Specimens were incubated in the primary antibodies acetylated alpha tubulin (Sigma, T6793), 3C11 (Developmental Studies Hybridoma Bank, SYNORF1), 5-HT (Sigma, S5545), and FMRFamide (Immunostar, 20091), and then diluted 1/200 in PBST (PBS containing 0.1% Tween 20) overnight at 4°C. After rinsing three times in PBST, samples were incubated with secondary antibodies labeled with Alexa 488, Alexa 568 (Molecular Probes, A11017 and A11036), and peroxidase (Sigma, A9917) diluted 1/200 in PBST for 1 h at RT. Samples were rinsed three times in PBST, mounted on slides in 50% glycerol in PBS, and observed under a confocal laser scanning microscope or a fluorescent microscope. For the peroxidase-conjugated secondary antibody, the chromogenic reaction was performed with 3,3′-diaminobenzidine (DAB) substrate.

### In situ hybridization

Animals were fixed with 4% PFA/MOPS buffer at 4°C for overnight and stored in 80% ethanol at −20°C. As for the same as antibody staining, whole animals and frozen sectioned (10 µm in thickness) samples were stained. After rehydration and three washes in PBST, samples were digested with 2 µg/mL Proteinase K/PBST for 15 min at 37°C. Following a brief wash with PBST, the samples were post-fixed in 4% PFA/PBST for 20 min at RT. After three washes in PBST, the samples were incubated in hybridization buffer (50% formamide, 5× SSC, 5× Denhardt's, 100 µg/mL yeast RNA, 0.1% Tween 20) for 1 h at 60°C and hybridized with DIG-labeled RNA probes at 60°C for at least 16 h. Excess probes were removed by washing the samples in 50% formamide, 2× SSC and 0.1% Tween 20 for 1 h; twice in 2× SSC and 0.1% Tween 20 for 30 min; and twice in 0.2× SSC and 0.1% Tween 20 for 30 min. Then the samples were incubated with a 0.5% blocking reagent in PBST for 1 h at RT. After blocking, embryos were incubated with alkaline phosphate-conjugated anti-DIG antibodies, and positive immunoreactions were visualized using nitro blue tetrazolium/5-bromo-4-chloro-3-indolyl phosphate (NBT/BCIP) solution (Roche). Then the samples were mounted in 50% glycerol/PBST or benzyl alcohol/benzyl benzoate and observed under a light microscope. The sequences for the probes were submitted to DDBJ. Accession numbers of *L. satsuma* genes are as follows: *elav*, AB715374; *syt*, AB715375.

### Scanning electron microscopy

Animals were fixed with 2.5% glutaraldehyde in ASW (artificial sea water) at 4°C. Samples were washed in ASW and post-fixed with 2% OsO_4_/ASW for 2 h at 4°C. After several washes with DW, samples were incubated with 1% aqueous tannic acid (pH 6.8) for 1 h for conductive staining. The samples were again washed with DW, treated with 1% OsO_4_/DW for 30 min and washed with DW. The samples were dehydrated in a graded ethanol series. After critical-point drying, the samples were coated with osmium. The coated samples were observed with a scanning electron microscope.

## References

[1] Cavanaugh CM (1985) Symbioses of chemoautotrophic bacteria and marine invertebrates from hydrothermal vents and reducing sediments. Bull Biol Soc Wash 6: 373–388.

[2] Cavanaugh CM, Gardiner SL, Jones ML, Jannasch HW, Waterbury JB (1981) Prokaryotic cells in the hydrothermal vent tube worm *Riftia pachyptila* Jones: possible chemoautotrophic symbionts. Science. 213: 340–342. 10.1126/science.213.4505.340 17819907

[3] Bright M, Lallier FH (2010) The biology of vestimentiferan tubeworms. Oceanogr Mar Biol Annu Rev 48: 213–266.

[4] Jones ML (1985) On the Vestimentifera, new phylum: six new species, and other taxa, from hydrothermal vents and elsewhere. Bull Biol Soc Wash 6: 117–158.

[5] Rouse GW (2001) A cladistic analysis of Siboglinidae Caullery, 1914 (Polychaeta, Annelida): formerly the phyla Pogonophora and Vestimentifera. Zool J Linn Soc 132: 55–80.

[6] Pleijel F, Dahlgren TG, Rouse GW (2009) Progress in systematics: from Siboglinidae to Pogonophora and Vestimentifera and back to Siboglinidae. C R Biol 332: 140–148. 19281947 10.1016/j.crvi.2008.10.007

[7] Rouse GW, Fauchald K (1997) Cladistics and polychaetes. Zool Scr 26: 139–204.

[8] Rouse GW, Goffredi SK, Vrijenhoek RC (2004) Osedax: bone-eating marine worms with dwarf males. Science 305: 668–671. 15286372 10.1126/science.1098650

[9] Rouse GW, Fauchald K (1995) The articulation of annelids. Zool Scr 24: 269–301.

[10] Kojima S, Hashimoto T, Hasegawa M, Murata S, Ohta S, et al (1993) Close phylogenetic relationship between Vestimentifera (tube worms) and Annelida revealed by the amino acid sequence of elongation factor-1 alpha. J Mol Evol 37: 66–70. 8360920 10.1007/BF00170463

[11] Kojima S (1998) Paraphyletic status of Polychaeta suggested by phylogenetic analysis based on the amino acid sequences of elongation factor-1 alpha. Mol Phylogen Evol 9: 255–261. 10.1006/mpev.1997.0472 9562984

[12] McHugh D (1997) Molecular evidence that echiurans and pogonophorans are derived annelids. Proc Natl Acad Sci U S A 94: 8006–8009. 9223304 10.1073/pnas.94.15.8006 PMC21546

[13] Black MB, Halanych KM, Maas PAY, Hoeh WR, Hashimoto J, et al (1997) Molecular systematics of vestimentiferan tubeworms from hydrothermal vents and cold-water seeps. Mar Biol 130: 141–149.

[14] Halanych KM, Feldman RA, Vrijenhoek RC (2001) Molecular evidence that *Sclerolinum brattstromi* is closely related to vestimentiferans, not to frenulate pogonophorans (Siboglinidae, Annelida). Biol Bull-US 201: 65–75. 10.2307/1543527 11526065

[15] Halanych KM, Lutz RA, Vrijenhoek RC (1998) Evolutionary origins and age of vestimentiferan tube-worms. Cah Biol Mar 39: 355–358.

[16] Richter S, Loesel R, Purschke G, Schmidt-Rhaesa A, Scholtz G, et al (2010) Invertebrate neurophylogeny: suggested terms and definitions for a neuroanatomical glossary. Front Zool 7: 29. 21062451 10.1186/1742-9994-7-29 PMC2996375

[17] Orrhage L, Müller MCM (2005) Morphology of the nervous system of Polychaeta (Annelida). Hydrobiologia 535/536: 79–111.

[18] Müller MCM (2006) Polychaete nervous systems: ground pattern and variations–cLS microscopy and the importance of novel characteristics in phylogenetic analysis. Integr Comp Biol 46: 125–133. 21672729 10.1093/icb/icj017

[19] Denes AS, Jékely G, Steinmetz PRH, Raible F, Snyman H, et al (2007) Melecular archtecture of annelid nerve cord supports common origin of nervous system centralization in bilateria. Cell 129: 277–288. 17448990 10.1016/j.cell.2007.02.040

[20] Tomer R, Denes AS, Tessmar-Raible K, Arendt D (2010) Profiling by image registration reveals common origin of annelid mushroom bodies and vertevrate pallium. Cell 142: 800–809. 20813265 10.1016/j.cell.2010.07.043

[21] van der Land J, Nørrevang A (1977) Structure and relationships of *Lamellibrachia* (Annelida, Vestimentifera). K Dan Vidensk, Selsk Biol Skr 21: 1–102.

[22] Jones ML, Gardiner SL (1989) On the early development of the vestimentiferan tube worm *Ridgeia* sp. and observations on the nervous system and trophosome of *Ridgeia* sp. and *Riftia pachyptila* . Biol Bull-US 177: 254–276.

[23] Gardiner SL, Jones ML (1993) Vestimentifera. In: Harrison FW, Rice ME, editors. Microscopic Anatomy of Invertebrates. New York: Wiley-Less, Inc. pp. 371−460.

[24] Karaseva NP, Malakhov VV, Galkin SV (2012) The morphology and anatomy of the vestimentiferan worm *Oasisia alvinae* Jones, 1985 (Annelida: Siboglinidae). II. Integument, nervous system and musculature. Russ J Mar Biol 38: 10–21.

[25] Webb M (1969) *Lamellibrachia barhami*, gen. nov. sp. nov. (Pogonophora) from the Northeast Pacific. Bull Mar Sci 19: 18–47.

[26] Jones ML, Gardiner SL (1988) Evidence for a transient digestive tract in Vestimentifera. Proc Biol Soc Wash 101: 423–433.

[27] Heuer CM, Müller CHG, Todt C, Loesel R (2010) Comparative neuroanatomy suggests repeated reduction of neuroarchitectural complexity in Annelida. Front Zool 7: 13. 20441583 10.1186/1742-9994-7-13 PMC2874545

[28] Heuer CM, Loesel R (2008) Immunofluorescence analysis of the internal brain anatomy of *Nereis diversicolor* (Polychaeta, Annelida). Cell Tissue Res 331: 713–724. 18071754 10.1007/s00441-007-0535-y

[29] Heuer CM, Loesel R (2009) Three-dimensional reconstruction of mushroom body neuropils in the polychaete species *Nereis diversicolor* and *Harmothoe areolata* (Phyllodocida, Annelida). Zoomorphology 128: 219–226.

[30] Loesel R, Heuer CM (2010) The mushroom bodies–prominent brain centres of arthropods and annelids with enigmatic evolutionary origin. Acta Zool 91: 29–34.

[31] Kaufman ZS (2008) Some problems of regressive evolution. Biol Bull 35: 318–326. 10.1134/s106235900803014x 18663975

[32] Holmes Bullock T (2002) Grades in neural complexity: how large is the span? Integr Comp Biol 42: 757–761. 21708772 10.1093/icb/42.4.757

[33] Müller MCM, Westheide W (2000) Structure of the nervous system of *Myzostoma cirriferum* (Annelida) as revealed by immunohistochemistry and cLSM analyses. J Morphol 245: 87–98. 10906744 10.1002/1097-4687(200008)245:2<87::AID-JMOR1>3.0.CO;2-W

[34] Hessling R (2003) Novel aspects of the nervous system of Bonellia viridis (Echiura) revealed by the combination of immunohistochemistry, confocal laser-scanning microscopy and three-dimensional reconstruction. Hydrobiologia 496: 225–239.

[35] Kristof A, Wollesen T, Wanninger A (2008) Segmental mode of neural patterning in sipuncula. Curr biol 18(15): 1129–1132. 18656359 10.1016/j.cub.2008.06.066

[36] Struck TH, Paul C, Hill N, Hartmann S, Hosel C, et al (2011) Phylogenomic analyses unravel annelid evolution. Nature 471: 95–98. 21368831 10.1038/nature09864

[37] Struck TH, Schult N, Kusen T, Hickman E, Bleidorn C, et al (2007) Annelid phylogeny and the status of Sipuncula and Echiura. BMC Evol Biol 7: 57. 17411434 10.1186/1471-2148-7-57 PMC1855331

[38] Rousset V, Rouse GW, Siddall ME, Tillier A, Pleijel F (2004) The phylogenetic position of Siboglinidae (Annelida) inferred from 18S rRNA, 28S rRNA and morphological data. Cladistics 20: 518–533. 10.1111/j.1096-0031.2004.00039.x 34892959

[39] Ivanov AV (1963) Pohonophora. Academic Press, London.

[40] Schulze A, Halanych KM (2003) Siboglinid evolution shaped by habitat preference and sulfide tolerance. Hydrobiologia 496: 199–205.

[41] Halanych KM (2005) Molecular phylogeny of siboglinid annelids (a.k.a. pogonophorans): a review. Hydrobiologia 535/536: 297–307.

[42] Schulze A (2003) Phylogeny of Vestimentifera (Siboglinidae, Annelida) inferred from morphology. Zool Scr 32: 321–342.

[43] Jennings RM, Halanych KM (2005) Mitochondrial genomes of Clymenella torquata (Maldanidae) and Riftia pachyptila (Siboglinidae): evidence for conserved gene order in Annelida. Mol Biol Evol 22: 210–222. 15483328 10.1093/molbev/msi008

[44] Shinozaki A, Kawato M, Noda C, Yamamoto T, Kobokawa K, et al (2010) Reproduction of the vestimentiferan tubeworm *Lamellibrachia satsuma* inhabiting a whale vertebra in an aquarium. Cah Biol Mar 51: 467–473.

